# Generalized Violence as a Threat to Health and Well-Being: A Qualitative Study of Youth Living in Urban Settings in Central America’s “Northern Triangle”

**DOI:** 10.3390/ijerph16183465

**Published:** 2019-09-18

**Authors:** Maria De Jesus, Carissa Hernandes

**Affiliations:** 1 School of International Service, American University, Washington, DC 20016, USA; 2 Center on Health, Risk, and Society, American University, Washington, DC 20016, USA

**Keywords:** generalized violence, youth, northern triangle, Central America, social determinants of health

## Abstract

El Salvador, Guatemala, and Honduras rank among the top 10 countries experiencing violence in the world, despite not being at war. Although there is abundant literature on generalized violence in this “northern triangle” of Central America as a driver of out-migration to the United States, very little is known about the perspectives and experiences of youth who do not migrate. This study aimed to elicit the emic perspectives of youth residing in the region on how the day-to-day generalized violence produces a pervasive threat to the overall health and human security of youth as well as the key protective factors and resiliencies at work. We conducted two separate waves of qualitative research in 2015 and 2018 over a 6-month period, which included 60 in-depth interviews and six focus groups among Salvadoran, Guatemalan and Honduran youth living in urban areas. Qualitative thematic analysis revealed two meta-themes: (1) ‘Lack of health,’ defined as not experiencing peace within the family, the community, and the country’ and (2) ‘Resilience.’ Thematic clusters that reflect the first meta-theme are: (1) violence as a common occurrence; (2) living in fear and insecurity; (3) victimization; and (4) lack of state protection and services. Thematic clusters for the second meta-theme are: (1) a positive future outlook and a commitment to education; (2) transnational and local family network support; and (3) engagement in community-based youth groups. To interpret the findings, we adopt the Latin American Social Medicine and Collective Health (LASM-CH) approach that prioritizes perspectives from the region. Generalized violence is conceptualized as a systemic phenomenon that is generated and reproduced through the complex interactions of structural inequities and unequal power relations. The findings of this study provide new insights into the implementation of a different approach to address the generalized violence, insights that may guide multi-sectoral health policies and interventions both in the region and transnationally.

## 1. Introduction

The “northern triangle” of Central America, comprised of El Salvador, Guatemala, and Honduras, historically, has historically undergone political instability, state repression, and sustained violence due to civil war (in Guatemala from 1960–1996 and in El Salvador from 1979–1992). Today, Central America is experiencing an epidemic of generalized violence—defined as widespread and large-scale violence that is affecting the civilian population at large. El Salvador, Guatemala, and Honduras consistently rank among the top 10 countries experiencing violence in the world, despite not being at war [1]. In El Salvador alone, 20,000 individuals were killed from 2014 to 2017 [2]. This figure amounts to more violent deaths than the number of deaths combined in several countries that were at war during those years such as Libya, Somalia, and Ukraine [2]. As a consequence, thousands of individuals and families have fled these countries and have moved northward to the United States seeking safety [3].

There is a mounting body of evidence specifically in the global public health literature linking war and other traumatic events to negative psychological and physical population health outcomes [4,5,6]. Victims of massive violent crimes experience post-traumatic stress disorder (PTSD) and other mental health issues [7,8,9,10,11]. Studies have also found some support for physical effects including a decrease in child and maternal health due to lack of resources and important nutrients when communities are experiencing security threats [6,12].

While interpersonal violence, armed conflict, and war have been studied extensively as determinants of mental and physical health outcomes [11,13,14,15,16,17,18], there are fewer studies that elicit the perspectives of those who are directly exposed to this violence. We argue that the scholarly discussion should not be limited to how generalized violence in Central America functions solely as a driver of out-migration. Instead, for a portion of youth who do not migrate, our study is essential to understanding how the day-to-day generalized violence produces a pervasive threat to the overall health and human security of youth as well as the key protective factors and resiliencies that have mitigated some of this threat. By adding the perspectives of this often-forgotten segment, we can better formulate policy and programmatic strategies in the region.

### 1.1. History of Violence in the “Northern Triangle” of Central America

Central America’s “northern triangle” has a long history of violence as evidenced by the unrest, prolonged civil wars, and military coups in El Salvador, Guatemala, and Honduras to the current context of generalized violence [19,20]. The long-running context of violence, including the influence of civil war, state and non-state violence, and the creation of gangs as imports from the United States, has permeated the region [19,21,22].

As Honduras, Guatemala, and El Salvador transitioned into fledgling democracies in the early 1980s, groups, such as the military, remained close to the levers of power formally or informally through the establishment of shadow and para-military networks closely connected to the government [20,23,24].

While Honduras did not experience a civil war like other countries in the “northern triangle,” it does have a history of political violence and effects from regional civil wars. Up until the early 1980s, the military of Honduras frequently ruled the country with support from elites [25]. During those periods and after transitioning to democracy, repressive tactics for controlling suspected Communist spies and others ideologically opposed to the government continued [26,27].

Guatemala experienced a 36-year civil war from 1960–1996. During the civil war, over 200,000 people were disappeared or killed while the military held power [28]. State-sponsored violence has been a marked characteristic of Guatemalan history continuing to the present [29], creating social insecurity along with drug-related violence and youth gangs [24,30,31]. In Guatemala, direct state-sponsored violence along with organized crime and gang violence is pervasive. Cocaine and heroin trafficked from South America to the United States make Guatemala a major transit country with the corresponding high levels of drug-related crimes [28,32].

El Salvador experienced a twelve-year civil war beginning in 1980 that claimed the lives of more than 75,000 people, left 8000 disappeared, and displaced one-fifth of the Salvadoran population [33].While there was a period of demilitarization and a peace process, the Farabundo Martí National Liberation Front (*Frente Farabundo Martí para la Liberación Nacional*; FMLN) continues to exist as a political party, and few strides have been made in economic justice [34]. The country continues to have high levels of inequality [35]. Additionally, while there was a Truth Commission to address wartime violence, war crimes were pardoned, and no reparations were made despite the Salvadoran state being named the main perpetrator of violence through its “systematic institutionalization of violence” [35]. Due to this lack of condemnation for past injustices and the active collaboration of civilians and private militia groups to enforce post-war order, the culture of state-sanctioned violence is particularly strong in El Salvador [24]. Organized crime, youth gangs, and street crime are pervasive forms of violence in El Salvador [24,36].

**Figure 1:**
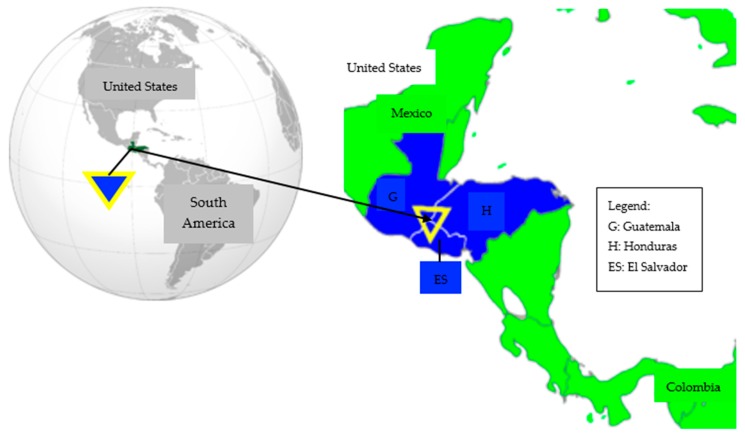
Map of Central America’s “Northern Triangle”. Source: Northern Triangle of Central America: Guatemala, Honduras, and El Salvador [39].

Central America’s predicament is one of a geography sandwiched between some of the world’s largest drug producers in South America and the world’s largest consumer of illegal drugs, the United States (Figure 1). The region is awash in weapons and gunmen, and high rates of poverty ensure substantial numbers of willing recruits for organized crime syndicates. Weak, underfunded, and corrupt governments struggle to keep up with the challenge. Though the United States has offered aid to address criminal violence, it also contributes to the problem through its high levels of drug consumption, relatively relaxed gun control laws, and deportation policies that have sent home more than a million illegal migrants with violent records. Currently, Honduras, Guatemala, and El Salvador are experiencing extreme levels of generalized violence that make it among the most dangerous countries in the world, especially for females, as demonstrated by increasing rates of violence against women and girls [28,37,38].

### 1.2. Gang Violence in El Salvador, Guatemala, and Honduras

Gang membership is a major risk factor among youth in El Salvador, Guatemala, and Honduras [40,41]. The extreme violence and illicit drug trafficking that now characterizes gangs in the “northern triangle” began in the inner cities of the United States as immigrant groups banded together for protection from other gangs [42]. For example, the Mara Salvatrucha (known as MS-13), now considered a transnational criminal gang, originated in Los Angeles in the 1970s and 1980s by Salvadoran immigrants in the city’s Pico-Union neighborhood who immigrated to the United States after the Central American civil wars. As their members spent time in the criminal justice system, beginning in 1990, many were deported to countries they hardly knew [42]. In order to survive and, in part, due to the lack of programs to receive them upon deportation, these returnees gravitated towards gangs bringing with them the drugs and violent practices they had learned in the United States [42,43]. Over time, these gangs grew into transnational criminal organizations.

The national, regional, and international efforts intended to curb violence in the “northern triangle” have not been very successful. Although enjoying public support, even militarized responses to criminal violence have not been successful. For example, in Guatemala, still scarred by a decades long civil war, guns and armed groups remain common. Since the early 2000s, “mano dura” (i.e., iron fist) policies and “tough on crime” approaches have enacted harsher punishments for gang members and expanded police powers, which contribute to increased levels of violence in the “northern triangle” of Central America, with community members witnessing an escalation of violence between gangs and the police [2]. These policies in most cases failed to reduce violence and crime and indirectly led to a growth in gang membership [2]. Mass incarceration increased the burden on already overcrowded prisons, where gangs, who often run the prisons, recruited thousands of new members [2]. Women and girls are increasingly targeted as recruits or victims of sexual violence and femicide [44].

### 1.3. Health Impact of Violence on Youth

Given that this study examines youth within a larger context of generalized violence, it is important to review why it is vital to study the health impact of violence on children and youth. Evidence from neuroscience research indicates that exposure to violent circumstances that produce persistent fear and chronic anxiety “predict significant risk for adverse long-term outcomes from which children do not recover easily” [45]. Behavioral neuroscience indicates that chronic activation of the stress response system leads to both immediate and long-term problems in physical health and mental health. Moreover, prolonged and/or excessive exposure to fear-inducing stimuli and stress (known as ‘toxic stress’) or threatening environments has been found to impair cognitive control and learning [46].

A wealth of literature, including work done by the Harvard University Center on the Developing Child [47], attests that children need safe, nurturing, and predictable environments to thrive [48]. Continuous, responsive interactions with caregivers shape neural development, influence attachment, and set the foundation for self-regulation skills. Simply put, feeling safe is fundamental for healthy youth development [49].

### 1.4. The Latin American Social Medicine and Collective Health (LASM-CH) Approach

In this study, we adopt the Latin American Social Medicine and Collective Health (LASM-CH) approach [50,51,52,53,54,55,56,57] to conceptualize how the phenomenon of generalized violence itself must be analyzed and addressed in relation to the adverse factors that generate and reproduce the violence in the first place. Generalized violence is viewed as a systemic phenomenon that stems from the complex interactions of structural inequities and unequal power relations. The LASM-CH approach recognizes that tackling the underlying social determinants of health is necessary (yet insufficient) to eradicating the generalized violence, which is a direct threat to the health and well-being of Central American youth. Furthermore, the approach pushes the ‘health agenda’ to go beyond addressing the social determinants of health as conceptualized by the World Health Organization [58]. It is a radical call to explicitly address the unequal power relations that drive the social determinants of health [59]. Furthermore, this approach conceptualizes health as a human right and is committed to the living conditions of the majority population [60].

From this perspective, the LASM-CH approach is a broader political, social, and cultural movement that prioritizes a broader conceptualization of health and solutions from and for the Global South [57,60]. This approach is particularly useful in conceptualizing the epidemic of generalized violence in the “northern triangle” of Central America as rooted in a larger socio-political context characterized by prolonged civil wars (in El Salvador and Guatemala), adverse colonial legacies, poverty among the majority population, unequal power relations, and wide social and health inequities. Study findings are interpreted using the LASM-CH approach, which provides new insights into the implementation of a different framework, insights that may guide multi-sectoral health policies and interventions to eradicate this direct threat to the health and well-being of Central American youth.

## 2. Methods

### 2.1. Research Design and Participant Selection

The study adopted an inductive, qualitative-driven research design, grounded in an emic or idiographic approach to research which “concerns itself with the specific and unique richness of a phenomenon” [61]. Eligibility criteria for participation included being a youth (i.e., between the ages of 15 and 24 as defined by the United Nations), having at least one close family member who had migrated to the U.S., and currently residing in an urban setting in Guatemala, El Salvador or Honduras.

The rationale for the eligibility criteria for this study is based on the conceptualization of a larger research program related to urban studies, migration, and health in the local and global contexts. The research program explores the perspectives of Central American youth in mixed-status families in the Washington, D.C. Metropolitan Area (where there is a large Central American population) as well as youth in the country of origin (i.e., Guatemala, El Salvador, or Honduras). The focus is on youth residing in urban areas where the violence is most prevalent and those who have at least one family member who has migrated to the United States to better understand the push and pull factors that drive some family members to migrate and others to stay behind in their countries, as well as how family members adapt locally and transnationally. The present study presents data from the perspectives of youth in the country of origin.

The average age was 18. Approximately half (52%) of the participants were male. Many of the participants (64%) were pursuing post-high school education. Prior fieldwork in this region on an earlier project facilitated the principal researcher’s entry for the qualitative research described here. Using a snowball sampling technique [62], the first author used community key informants to purposefully select an initial sample of participants who met eligibility criteria. These participants then nominated, through their social networks, other participants who met the eligibility criteria. This sampling technique is often used in hidden populations, which are difficult for researchers to access given that there are no lists or other obvious sources for locating members of the population of specific interest [62].

### 2.2. Data Collection and Analysis

Data were collected through open-ended semi-structured in-depth interviews and focus groups during two waves of data collection from June to August in 2015 and 2018, respectively. The first author conducted 60 hour-long in-depth interviews (20 in Guatemala, 20 in El Salvador, and 20 in Honduras, respectively) and six 90 min focus groups (i.e., two in each country, respectively) in Spanish. The interviews and focus groups were held in private spaces in convenient community-based sites. The purpose of the study was clarified, and informed oral consent was obtained from all participants. All interviews and focus groups were recorded, and analytic memos were created following each interview and focus group [62,63]. All participants received a $10 USD incentive.

Data collection and analysis was an iterative process. Following each interview and focus group, the first author listened to the recording and began analyzing the data by documenting salient themes and patterns both within each interview/focus group and across interviews/focus groups. Data saturation was achieved with the sample as there was no new information that emerged with new participants. Approval from the University’s Institutional Review Board was received prior to conducting the field research.

To analyze the data, both authors conducted a qualitative thematic analysis using ATLAS.ti (V.7.0), (Scientific Software Development, Berlin, Germany), which allowed us to search for meanings at the level of coding and for contextualized understandings within the data at the level of interpreting results [64,65]. Stability and agreement are the most relevant types of reliability for textual data. Following category revision, an interrater reliability of 96 per cent was achieved. To see if this agreement was due to chance, the intercoder reliability was tested using Cohen’s Kappa [66]. The overall Kappa coefficient was 0.97. We generated codes based on the data themselves and developed a codebook that was modified as needed during the analytic process [64,65,67]. Through an interpretive process, we located patterns in the codes and collapsed codes into themes (i.e., data reduction). In the first level of thematic analysis, descriptive subthemes were developed by reducing the number of categories and looking for patterns across categories. Subthemes were vivid descriptions that evoked the essence of all of the participants’ experiences and preserved the affective tone of their words. Descriptive subthemes were then subsumed under abstract clusters of themes (second-level thematic analysis). Finally, these clusters were categorized under a meta-theme (third-level thematic analysis). The data were then visually displayed in tables and a figure to facilitate comparison and interpretation of themes. The first author also conducted member-checks to ensure validity and accuracy of the findings.

## 3. Results

**Figure 2:**
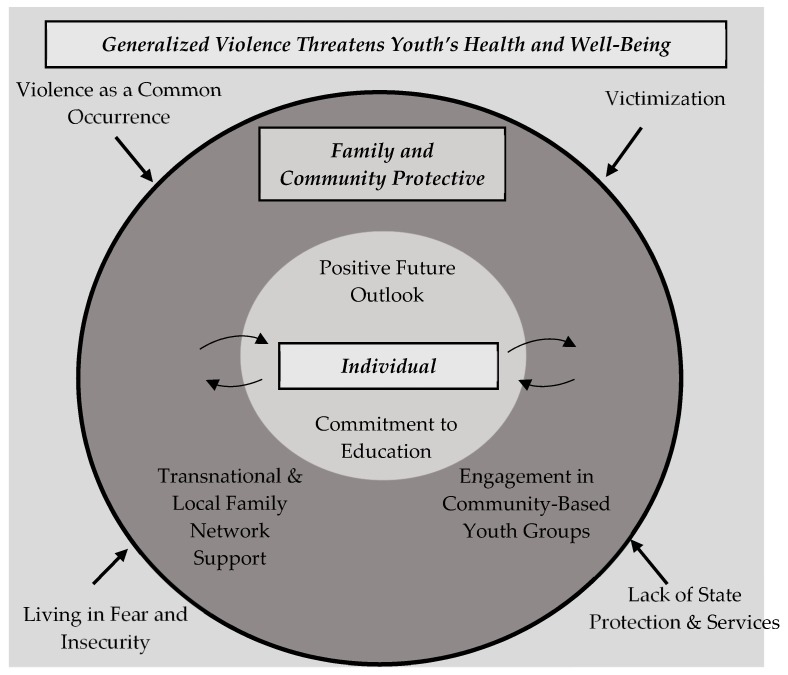
Conceptual Model: Generalized Violence, Family and Community Protective Factors, and Individual Resilience.

We identified two meta-themes in the study: (1) ‘Lack of health,’ defined as ‘not experiencing peace within the family, the community, and the country’ and (2) ‘Resilience.’ The respective thematic clusters and subthemes reflect the experiences of the majority of the youth focus group participants and the interviewees and are reported below (Table 1; Table 2; Figure 1, Figure 2). Pseudonyms are used for all the participants.

### 3.1. Meta-Theme 1: Lack of Health, Defined as Not Experiencing Peace Within the Family, the Community, and the Country

All of the focus group participants and interviewees spoke about the intersections of everyday violence and the importance of understanding its multi-level effects on overall health and well-being.

As one focus group participant stated:

We experience and see violence everywhere. Health is not only concerned with what is happening with an individual in terms of psychological or physical health problems. Health also encompasses what is happening at the family, community, and country level. Health means experiencing peace within our family, our community, and our country. Health is peace and overall well-being. We do not experience health here. (Coralie, Guatemala City)

#### 3.1.1. Cluster 1: Violence as a Common Occurrence

The first thematic cluster refers to subthemes that depict the day-to-day occurrence of generalized violence all around them.

***Massacred bodies on the streets.*** The majority of the focus group participants and interviewees spoke about the violence that they had witnessed in the streets or their neighborhoods. Lucia, a female interviewee from San Salvador, stated: “You get more sympathy from seeing a dead animal than a dead body. It is the norm here to see dead bodies on the streets or see the violence in the news everyday.” Similarly, another interviewee stated: “The first thing I saw from the bus on a main highway going into Tegucigalpa in the early morning was a body being put in a black body bag. The massacred body had been disposed of in the middle of the highway. As the bus driver described, ‘It was deposited there most likely to send a clear message by the *maras.*’”

***Rival gangs kill each other and other victims.*** All of the participants commented on the pervasiveness of gang-related violence. Aron, a male focus group participant from San Salvador, recounted: “Males in particular have a difficult time. There are two main rival gangs: *Mara Salvatrucha MS13* and *MS18*. They cannot go into the wrong neighborhoods. It is designated by the colors that they wear. They also wear sports teams clothing. At one point, the word on the streets was that people wearing certain colors would get killed.” Tomas, another focus group from San Salvador, described a recent gang-related event that had made recent local news: “There was a kid who the *pandilleros* threw over a bridge. He landed on a makeshift structure of homeless people who were living under the bridge. The boy ended up in the hospital and died a few days later. The *pandillas* wanted the boy to kill a bus driver and he refused; so they used him to set an example.”

Gang recruitment was a salient topic that frequently emerged across all of the interviews and the focus groups. Carlos, an interviewee from Guatemala City, recounted:

These young kids around here are being recruited by the *pandillas* [gangs] to kill people who the *pandilleros* [gang members] are extorting. Not too long ago, the *pandillas* were extorting a bus driver who did not pay up. They asked a kid from the community to go and kill this man. They then gave him 150 *quetzales*. The kid brought the money to his grandmother and she asked where he got this money. The kid responded that it was for some work he had done for a couple of men. Meanwhile the work is killing. These kids end up becoming *pandilleros* themselves.

***Police-gang killings.*** The majority of the focus group participants and interviewees also described the growing violence between the police and gangs. Juan, a focus group participant from San Salvador, described: “There is a lot of violence now between the police and gang members. If gang members see that they [police officers] are part of the PNC [Policia Nacional Civil], they kill them. There was a story in the newspaper where a PNC was killed recently by “*mareros*” [gang members] presumably because he was a police officer.”

***Girls and women disappeared or are recruited by gang members*.** Most of the participants also highlighted how the violence is increasingly affecting girls and women in the community. Dolores, an interviewee from Tegucigalpa, stated: “There is more and more violence towards women. Women and young girls are disappearing. I am so afraid that I tell my sister to go to school and just come straight home.” Isaura, a focus group participant from San Salvador, referred to the gang recruitment: “Gangs members outside recruit women to go to jail and bring contrabands like cell phones, drugs and weapons to those on the inside. Young women are now affiliated with *pandillas*.”

#### 3.1.2. Cluster 2: Living in Fear and Insecurity

The second cluster refers to subthemes that describe the constant fear and insecurity the youth experience, which contributes to a ‘context of risk’ and affects the youth’s overall sense of health and well-being.

***Something bad will happen at any time.*** All of the focus group participants and interviewees shared the vulnerability they felt every time they left their homes. Ana, a focus group participant from San Salvador, stated:

You always have to be careful. Yes, there’s a feeling of constant insecurity because you live here and there is so much violence. My sister worries a lot and is very afraid. She’s so traumatized. I feel that she sees bad things everywhere; and in her mind something bad will happen at any time. She demands that I always take the same route and that I go from our home to the university and the university back home. I can’t go anywhere else.

Another female focus group participant from San Salvador, Carlota, shared similar sentiments: “I’m very scared and paranoid, I’m always looking around and very alert. One time I was with my friend on the bus and some people came in and I’m like, ‘Lay low! Lay low!**’** I squeezed my friend’s hand so hard it almost broke, but we got to the university safely, nothing happened.”

***Scared to go anywhere especially outside my neighborhood.*** Similarly, all of the participants expressed the fear that they felt anytime they traveled outside their neighborhoods. Gisela, an interviewee from San Salvador, described:

I am scared to go anywhere, especially outside my neighborhood. There are places that are more dangerous for men than for women, because they’ll ask a man for their ID to see where he’s from. There are certain zones we cannot go into such as San Bartolo, Santa Marta, and Chácara Sierra Morena. We can’t go there because rival gangs live there. There are also divisions internally too. They are divided into territories inside their own groups.

***Culture of silence, mistrust, and fear.*** Most of the focus group participants and interviewees spoke about feeling afraid, which contributed to the silencing of the crimes and injustices they endured or witnessed in their day-to-day lives. As Ronaldo, an interviewee from Tegucigalpa, expressed: “There is a culture of mistrust and fear here. We are all afraid. Someone you know could be informing the ‘*maras’*.” Similarly, Rosa, a Guatemalan interviewee, stated: “We live in a culture where no one denounces the members. There is a culture of silence. We are afraid for our lives. We know gang members who live in front and beside us in the same neighborhood.”

#### 3.1.3. Cluster 3: Victimization

The third cluster refers to subthemes that reveal how youth are the victims of different forms of violence in the context of their daily lives, which affects their health and well-being.

***Getting extorted and threatened by gang members.*** The majority of the participants had either been directly extorted or threatened by gang members or knew of someone in their families who had been. Jose Luis, an interviewee from Guatemala City, shared his story of being a victim of gangs: “I used to work for a company selling cell phones. I had to stop because I was getting extorted by ‘*las maras’* [gangs]. I had to give them a percentage of the money I had made with sales. When I went to my boss and told him I did not have the entire sum of money from sales due to the gang members, my boss told me that I should not have given them the money.” Miguel, a focus group participant from San Salvador, described how his brother and family were also victims of gang activities:

My younger brother who is 15 years old was being recruited by the ‘*maras.*’ He refused and the ‘*mara’* got a hold of our house number and started calling with threats that they would kill him and our family. We were all afraid for his life. We were desperate. We sent him across the border illegally for his protection. He has been gone for one year and lives with our father in New Jersey.

***Getting robbed is the norm.*** All of the focus group participants and interviewees expressed that they had been victims of robbery involving force, intimidation, and/or coercion. Joanna, an interviewee from Tegucigalpa, recounted:

I have been robbed so many times. I do not know anyone who has not gotten robbed. You shouldn’t go anywhere in buses…especially alone with few people around, that’s almost a guarantee that you will be robbed. One time I sat by the window and immediately started to study. This guy sat by me and had his arm in a cast. He smelled of booze so bad, then he demanded my cell phone. From his cast, a knife came out. I had to give him my cellphone.

#### 3.1.4. Cluster 4: Lack of State Protection and Services

The fourth cluster refers to subthemes that demonstrate how youth are not protected by the state, which contributes to a ‘context of risk’ and threatens their sense of health and well-being.

***No security from the state and the police.*** All of the participants shared that they did not feel that the state provided them with a sense of security or protection. As Sofia, a focus group participant from San Salvador, stated: “We have no security from the state. We pay a tax to the government here for *‘nuestra seguridad’* [our security] but we do not feel safe. We pay the *‘maras’* too who want a cut. We have no safety here!” Flora, an interviewee from Guatemala City also conveyed how the police did not help the community feel secure: “The cops are never there when you need them. If there is an emergency, gunshots for example, the cops wait until the ambulance gets to the scene, and when all is said and done, they then create a show like they are actually investigating and reacting. It is all a performance.”

***No health and social services***. All of the focus group participants and interviewees spoke about the lack of health and social services in their countries. They also emphasized that the scarce resources (e.g., state-funded hospitals) that did exist were of poor quality, including not being staffed by well-trained personnel. Carlos, an interviewee from San Salvador, stated: “No one here goes to the hospital unless they are in extremely dire need of health care.”

***Fragmented families and children are on their own.*** All participants also recounted experiences related to the negative effects of violence, including family fragmentation and separation and the vulnerability of youth. As Cristiana, a Guatemalan interviewee, described: “In terms of kids who are crossing the border on their own: many times the grandmother dies and there is no one that can take care of the child financially. Other family members send them to the U.S. with a coyote so that a family member in the U.S. can take care of them. Many times the coyote abandons the child along the way…These vulnerable children are not being protected.”

***Children ripe for recruitment.*** The majority of the focus group participants and interviewees also spoke about younger children in their own families or in other families who had been approached by gang members with the purpose of recruiting them. Antonio, an interviewee from Tegucigalpa, stated: “Many of these kids live with grandmothers; many of their parents are living in the US now or are in jail or are lost to alcoholism. The *pandillas* come around and recruit these young kids.”

The second meta-theme, ‘Resilience’ refers to the protective-stabilizing factors that Central American youth exhibit in their lives, which mitigate the threat of violence on their health and well-being and, ultimately, deter them from turning to violence themselves. The respective thematic clusters and subthemes that were identified are reported below (Table 2).

### 3.2. Meta-Theme 2: Resilience

All of the focus group participants and interviewees described resiliencies, such as remaining optimistic about the future, a commitment to education, the importance of the social and financial support from family members, as well as their engagement with youth groups in the community.

All of the participants expressed a strong sense of obligation to do well, especially given that their family members in the U.S. and in the country were offering them support and making a financial and emotional sacrifice for them to succeed. Franci, a focus group participant from Tegucigalpa, stated: “My mother who is in Maryland sends money every month to help us. She offers me a lot of support and my older sister here does too. I will succeed. My family is making the ultimate sacrifice for me and I need to make sure I succeed so that their sacrifice is worth it. I want my mother to be proud of her son who is here.”

The first thematic cluster refers to subthemes that depict the youth’s optimism for a better future and focus on school success.

#### 3.2.1. Cluster 1: Positive Future Outlook and Commitment to Education

***I want to do well in school to have a good future.*** All of the participants shared a strong desire to succeed in school with an aim to have a better future for themselves. Alma, an interviewee from San Salvador, recounted: “I am studying anesthesiology at the public university and want to succeed. I want to stay here and have a career here. I want to be part of the professional middle class in my country and have a good future. I do not want to go to the U.S. and do service jobs.”

***I want my country to progress.*** Beyond their own individual success, all of the participants also expressed a desire for positive change in their country. Cristiana, an interviewee from San Salvador stated: “We need to think about what’s going to happen to our country if everybody leaves. There will be no doctors, no economists, etc. here. How can our country make any progress if there aren’t any people who can be here and work for the country’s future? I want to see my country’s future be stronger and better.”

#### 3.2.2. Cluster 2: Transnational and Local Family Network Support

The second cluster refers to subthemes that depict the different forms of support the youth receive from transnational and local family members.

***Family members here and in the United States offer me social and emotional support.*** All of the focus group participants and interviewees reported receiving social support from their family members in the United States as well. Participants communicated regularly with their family members in the U.S, via phone or text messaging. There were also family members who served as surrogate parents in the home country. Family social support networks across and within borders were complex. For example, Alma, an interviewee from San Salvador, stated: “My older sister is here and she is a mother figure to me. Our mother supports us financially and emotionally from the U.S.”

***Family members in the United States offer me financial support for school expenses and for our family needs here.*** All of the participants also received remittances from family members who had migrated to the U.S., which permitted them to stay in their home countries and pay for school expenses and for other services and household needs. Sonia, a focus group participant from Guatemala City, described: “I have a brother in Chicago. He went as a ‘*mojado.’* (i.e., ‘wetback’: a slang term that refers to one who crosses an international border by passing through a body of water by swimming from the shore of one country to enter the adjacent country without any legal documents). He left his wife and kids behind and now has another wife, a Salvadoran, and 3 other kids in the U.S. He sends them money more than once a month and when there is an emergency he helps them out monetarily. He helps me too.”

#### 3.2.3. Cluster 3: Engagement in Community-Based Youth Groups

The third cluster centers on subthemes that demonstrate how the youth’s active participation in and support received from community-based organizations helps buffer against the negative consequences of the violence and insecurity they experience.

***By actively participating in the youth group, I feel like I am contributing to my community.*** A majority of the participants were members of a youth group and spoke positively about belonging to such a group. Coralie, a Guatemalan participant, stated: “When I come here [youth group] and get involved in projects that will improve our community, I know I am doing good. Similarly, Gustavo from San Salvador conveyed a similar perspective: “Together, through the community work we do with *Centro Bartolomé de las Casas*, we are making the community better.”

***I get social support from my peers in the youth group.*** The majority of the participants also elaborated on the benefits of staying involved in the youth group. Isaura from San Salvador recounted: “I can always count on my friends here. This group helps me, especially when I am down about what is happening in our community.” Berta from Tegucigalpa also shared the importance of receiving social support from the youth group: “These individuals here [points to those around her] are incredible positive forces in my life and help me to keep moving forward.”

## 4. Discussion

This study makes an important contribution to the social science literature at the nexus of urban studies, migration, and health. It sheds light on the generalized violence in the “northern triangle” of Central America as well as the resiliencies and protective factors from the perspectives of the youth themselves who live in this region. As revealed in the first meta-theme and clusters of subthemes, generalized violence is a common occurrence for the youth in the region. They experience violence, insecurity, and victimization as a daily reality of life. Coupled with the lack of state protection and social and health services, youth in the “northern triangle” exist within a threatening environment. The participants described peer, family, and community support, a positive outlook about the future, and commitment to education as the primary motivators in their lives. These findings are consistent with other studies on youth and violence in other parts of the world [16,68,69].

The participants viewed generalized violence as a powerful determinant of their health and well-being at multiple nested levels. For example, at the individual level, they experience fear and insecurity, especially traveling outside their homes and/or neighborhoods, which leads to the silencing of the crimes and injustices they endure or witness in their daily lives. At the relationship and community levels, families are fragmented and separated due to the generalized violence.

Resiliencies and protective factors were also evident at multiple levels. At the individual level, youth experienced a commitment to education and a positive outlook on the future. At the relationship level, they received support from their friends and relatives in-country and transnationally. At the community level, youth were engaged with and drew support from their peers at the community-based youth groups to which they belonged. Although the youth interviewed in this study have not chosen to migrate and are not directly involved in the violence, this does not mean those protective factors are guaranteed or that their situations will not change, especially given that the root factors (i.e., structural inequities and unequal power relations) that give rise to the generalized violence and adverse living conditions remain unchanged.

It is, therefore, critical that we adopt the Latin American Social Medicine and Collective Health (LASM-CH) approach [50,51,52,53,54,55,56,57] to interpret our findings and to conceptualize how the phenomenon of generalized violence itself must be analyzed and addressed in relation to the root factors that produce and sustain the violence. Based on this approach, we need both a broader conceptualization of health and solutions from and for the Global South [57,60]. Specifically, we must not simply address the social determinants of health as conceptualized by the World Health Organization [58], but also transform the unequal power relations that drive the social determinants of health [59] in the first place.

A LASM-CH approach, which prioritizes perspectives from the region [50,51,52,53,54,55,56,57], is also useful to translate the findings into multi-sectoral policies and interventions with the aim of creating widespread and long-lasting change in the region. This approach encompasses the broad context of health as a human right, including addressing social determinants, improving the living conditions of the majority populations, *and* ensuring the equitable distribution of health knowledge, resources, and services. From this perspective, isolated policy decisions and programs that target one sector or set of actors are unlikely to produce widespread change. Health, social, economic, and immigration actions are intrinsically related and thus, change in one domain affects others. There is a great need, therefore, for the development of comprehensive public policies in the “northern triangle” of Central America to eradicate the generalized violence, promote collective health and well-being as well as foster sustainable development and democratic progress in the region.

Our findings also show that within the context of the “northern triangle,” it is not only support within the local community or family that is important, but due to the history of migration, there is a strong transnational aspect to most relationships. These relationships include parents, siblings, and others who have migrated, mostly to the United States, in order to provide these youth with money and the stability it can bring; youth identified a desire to make their transnational supporters proud or to not waste the sacrifice they made. While these ties do not insulate youth from all risk factors (experiencing violence, threats, etc.), they provide support and motivation as expressed by the youth themselves. Future policies and programming should capitalize on these ties of social support at the family and community levels to ensure that the youth continue to thrive and to deter them from entering the cycle of violence themselves. It is important, however, to emphasize that promoting social support from family and community organizations are not the panacea for addressing the generalized violence in the region. While promoting social support for the youth is key, we also need to address the structural inequities and unequal power relations in order to eradicate the violence and unfavorable living conditions, as above-mentioned.

It is noteworthy that our findings on how transnational family support is critical to youth’s ability to remain in their countries of origin provides an interesting counter-perspective to research with Central American youth in the U.S. Many of the U.S.-based studies [70,71,72,73] demonstrate the exact opposite—that transnational family members were the principal protagonists in the decision-making process around whether and when youth migrated. One explanation for this discrepancy is age. Our interviews were conducted with older youth whereas the samples in U.S.-based studies comprise younger youth (e.g., ages 11–17). The divergence in youth experiences may suggest greater agency among late-adolescent youth and young adults in deciding whether to reunite with family in the U.S.

Another possibility likely relates to differences in caregiver arrangements. Among youth migrants in the U.S., a large number had migrated following the illness or death of a grandparent caretaker. Older youth, however, like those in our study, are likely better situated to fend for themselves in such circumstances without having to relocate abroad. Another equally plausible explanation is related to youth and their families’ (mis)perceptions regarding the possibility of securing legal status in the U.S., which is also tied to age. Youth ages 18–24 undoubtedly have far less possibility of entering or legally remaining in the U.S. than their younger counterparts and thus are not similarly persuaded to leave home. 

### Study Limitations and Strengths

Although this study exemplified the potential of an inductive, qualitative-driven research design, grounded in an emic or idiographic approach to analyze the perspectives of Central American youth, the study sample is not representative of all youth. Our sample comprised youth who lived in urban settings. Furthermore, many of the participants were pursuing post-secondary education, which is not representative of all Central American youth. Although we recognize that the sample is not generalizable to all youth in the “northern triangle’ of Central America, we provide a rationale for the eligibility criteria in the Methods section.

Despite this limitation of generalizability, our study makes a unique contribution to the literature given that it foregrounds the perspectives and experiences of Central American youth who have *not* migrated to the U.S. There is a plethora of studies on the perspectives and experiences of Central American youth who migrate to the U.S. [70,71,72,73], yet less is known about those who do *not* migrate. The decision to migrate is a complicated calculus and relies on factors on both micro and macro levels [74,75]. Our study adds another dimension to this discussion by capturing the voices of youth who do not migrate, voices that are equally as important.

## 5. Conclusions

This qualitative study has shown us the importance of interviewing those who have stayed in the region despite the logistical challenges of conducting research in these high-risk contexts. In order to craft effective and long-lasting change in the region through policy and program development, an in-depth understanding of the landscape of fear and insecurity that youth and other populations experience as well as the resiliencies and protective factors currently at work is key.

All future policy decisions, programs, and initiatives must be grounded in the reality of those who are directly affected by the violence. The inclusion and empowerment of local youth, community actors, and leaders is essential for health interventions to successfully and sustainably address the generalized violence. Once a richer understanding and coalition have been created, widespread change and commitment is needed to address the complex network of factors creating this context of generalized violence including poverty alleviation, federal and local governmental reform, police re-training, and rehabilitative and work training programs to transition and reintegrate gang members, many of whom are youth, into the legal economy.

Moreover, given the transnational nature of the gangs themselves and the effects of the generalized violence that drive northward migration, it is also important to interpret these findings in light of the potential implications for coordinated efforts between North and Central America. As such, there is a need to develop coordinated cross-border government policies that center a health-in-all-policies approach, as well as transnational research and initiatives that can contribute to long-term, sustainable solutions that address the generalized violence in Central America. The changing nature of violence itself in the region, from civil war historically to the present context of generalized violence, means we need to utilize different models and approaches to address the issue.

In the “northern triangle,” a perpetual cycle of generalized violence has been part of the daily reality of youth as findings in this article demonstrate, and this violence has amplified a highly uncertain, unsafe, and psychologically and physically threatening environment for these Central American youth and their families. A broad-based, multisectoral, and determined political resolve in both Central and North America is needed to put an end to this cycle.

## Figures and Tables

**Table 1:** An overview of descriptive subthemes (first-level thematic analysis), thematic clusters (second-level thematic analysis) and first meta-theme (third-level thematic analysis).

Meta-Theme 1: Lack of Health, Defined as not Experiencing Peace within the Family, the Community, and the Country			
Thematic Clusters: Violence as a Common Occurrence	Living in Fear and Insecurity	Victimization	Lack of State Protection and Services
*-Massacred bodies on the streets* *-Rival gangs kill each other and other victims* *-Police-gang killings* *-Girls and women disappeared or are recruited by gang members*	*-Something bad will happen at any time* - *Scared to go anywhere especially outside my neighborhood* *-Culture of silence, mistrust, and fear*	-*Getting extorted and threatened by gang members* *-Getting robbed is the norm*	-*No security from the state and the police* -*No health and social services* -*Fragmented families and children are on their own* -*Children ripe for recruitment*

**Table 2:** An overview of descriptive subthemes (first-level thematic analysis), thematic clusters (second-level thematic analysis) and second meta-theme (third-level thematic analysis).

Meta-Theme 2: Resilience		
Thematic Clusters: Positive Future Outlook and Commitment to Education	Transnational and Local Family Network Support	Engagement in Community-Based Youth Groups
*-I want to do well in school to have a good future* *-I want my country to progress*	*-Family members here and in the United States offer me social and emotional support* *-Family members in the United States offer me financial support for school expenses and for our family needs here*	*-By actively participating in the youth group, I feel like I am contributing to my community* *-I get social support from my peers in the youth group*
